# Rhabdomyosarcoma With Diffuse Bone Marrow Metastases

**DOI:** 10.7759/cureus.21863

**Published:** 2022-02-03

**Authors:** Daniel Huang, Pankaj Watal, Dennis Drehner, Deeksha Dhar, Tushar Chandra

**Affiliations:** 1 Radiology, University of Central Florida College of Medicine, Orlando, USA; 2 Radiology, Nemours Childrens Hospital, Orlando, USA; 3 Pathology, Nemours Children's Hospital, Orlando, USA; 4 Medicine, Government Medical College and Affiliated Hospitals, Jammu, IND; 5 Pediatric Radiology, Nemours Children's Hospital, Orlando, USA

**Keywords:** diffuse marrow infiltration, myeloproliferative, pediatric, predominant bone marrow metastases, fdg pet-ct, alveolar rhabdomyosarcoma

## Abstract

Rhabdomyosarcoma is a highly aggressive cancer that is generally considered a disease of childhood. A vast majority of cases occur in those below the age of 20. Rhabdomyosarcoma can occur in any soft tissue in the body but is primarily found in the head, neck, orbit, genitourinary tract, genitals, and extremities. Prognosis is closely tied to the location of the primary tumor and the extent of metastatic spread. As with most sarcomas, rhabdomyosarcoma has a pattern of hematogenous spread which favors metastasis to the lungs. Other common areas include bone marrows, liver, breasts, and brain. One unusual pattern is the presence of diffuse bone marrow metastases in absence of significant soft tissue disease other than primary (no distant nodal disease, absence of visceral disease in chest and abdomen). Frequently in such cases, patients may have initial presentation similar to hematologic malignancy especially when the primary tumor is not evident. This pattern has been rarely described in the radiology literature. This pattern appears to be well documented in pathology literature. Even more rarely, in some cases, the primary tumor site may not be found after imaging and may remain undetermined even postmortem - only diagnosed by bone marrow aspiration. Awareness of this unique pattern is clearly important for radiologists, especially pediatric radiologists, as misdiagnosis can lead to delay in appropriate treatment that ultimately results in increased mortality. We present a case of rhabdomyosarcoma with this unique pattern of bone marrow metastases in which initial differential diagnosis favored a leukemic picture. This paper will go over the diagnostic techniques utilized throughout our patient’s disease course as well as treatment.

## Introduction

Pediatric soft tissue sarcomas (STSs) are a rare group of tumors that originate from a primitive mesenchymal cell. They account for approximately 3%-4% of all childhood cancers in the United States at an estimated incidence of five per million. Rhabdomyosarcomas (RMSs) are the most common STS of childhood and represent approximately half of all STS in patients below 20 years of age [1]. These can present anywhere in the body, however, there are distinct patterns that link primary location, histology, and metastatic spread at the time of diagnosis. In the adolescent age group, extremity tumors are more common and are frequently of the alveolar type [1]. This subtype requires a predominant (>50%) alveolar component and a FOX01 rearrangement by FISH or RT-PCR per recommendation from the Soft Tissue Sarcoma Committee of the Children’s Oncology Group’s most recent guideline [2]. RMS with bone marrow (BM) metastasis accounts for approximately 6%-16% of all RMS cases as part of widespread disease. However, BM is rarely the sole metastatic site [1,3-7]. In this paper, we present an unusual case of primary alveolar RMS (ARMS) of the right lower extremity with the initial presentation of diffuse BM metastases as the sole metastatic site.

## Case presentation

**Figure 1:**
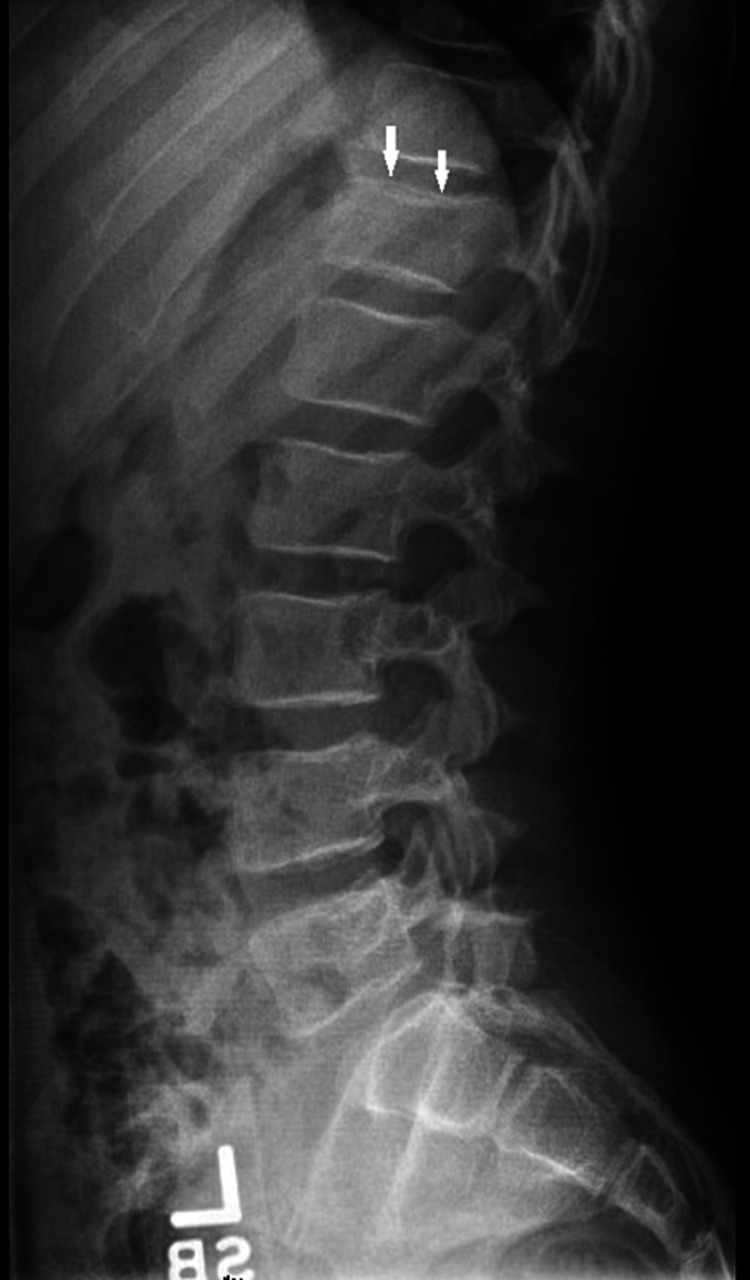
Lumbosacral spine, lateral view. Depression of the superior endplate of the T12 vertebra with wedging suggests a compression fracture (white arrow).

An 11-year-old male presented in the emergency department (ED) for increasing levels of back discomfort after a fall a few days prior to presentation. Thoracic and lumbar spine films were obtained revealing loss of vertebral body height involving T12 concerning compression fracture (figure 1). The patient’s pain did not subside with standard treatment. Subsequently, he presented in the ED after developing bruising to the legs, left leg pain, and right foot pain and swelling. He denied any trauma to the legs that would potentially cause bruising and had no complaints of discrete foot mass. No focal mass was evident to any of the clinical teams on examination. On further workup, he was found to have anemia, thrombocytopenia, and hypercalcemia. On imaging, an x-ray of the foot revealed diffuse soft tissue swelling but no acute fractures or malalignment. An ultrasound of the lower extremity was ordered to rule out deep venous thrombosis and found to be negative. Based on the clinical features and lab findings, BM pathology or infiltrative process such as leukemia was concerning, and BM biopsy was recommended.

**Figure 2:**
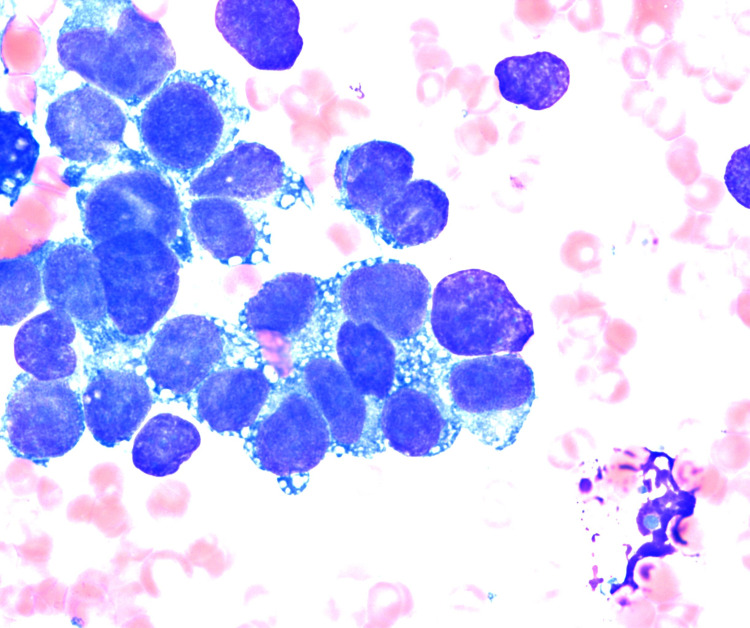
Wright Giemsa-stained aspirate smear showing a cohesive cluster of pleomorphic tumor cells (1000x).

**Figure 3:**
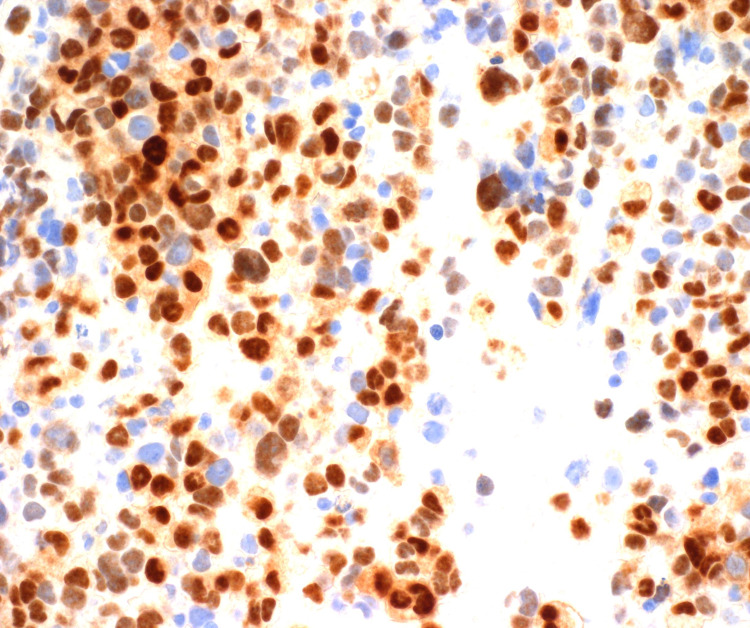
Strong nuclear staining for myogenin, a transcriptional regulatory protein expressed early in skeletal muscle differentiation. It supports a diagnosis of rhabdomyosarcoma.

BM aspirate smears had occasional cohesive clusters of cells scattered among normal hematopoietic precursors (Figure 2). The cells were large, ovoid, pleomorphic, and had primitive nuclear chromatin. Flow cytometry revealed a CD45-negative, CD56-positive population at 74% of nucleated cells. CD45 is a hematopoietic cell marker and CD56 is a marker positive in many non-hematopoietic neoplasms. These findings suggested a non-hematopoietic neoplasm metastatic to BM. Immunohistochemical assays for desmin, myogenin, CD56, and vimentin were positive in the tumor cells (Figure 3). Cytokeratin, CD31, synaptophysin, NKX2.2, WT1, and Melan-A were negative. INI-1 was expressed ruling out the rhabdoid tumor. The histologic findings were consistent with rhabdomyosarcoma. FOX01(13q14) FISH on the clot section was positive indicating a rearrangement involving the FOX01 gene region, a finding associated with ARMS. This was confirmed by chromosome analysis on the aspirate which showed near-tetraploid karyotype with complex chromosomal abnormalities including t(2;13)(q36;q14) and the presence of double minute structures.

**Figure 4:**
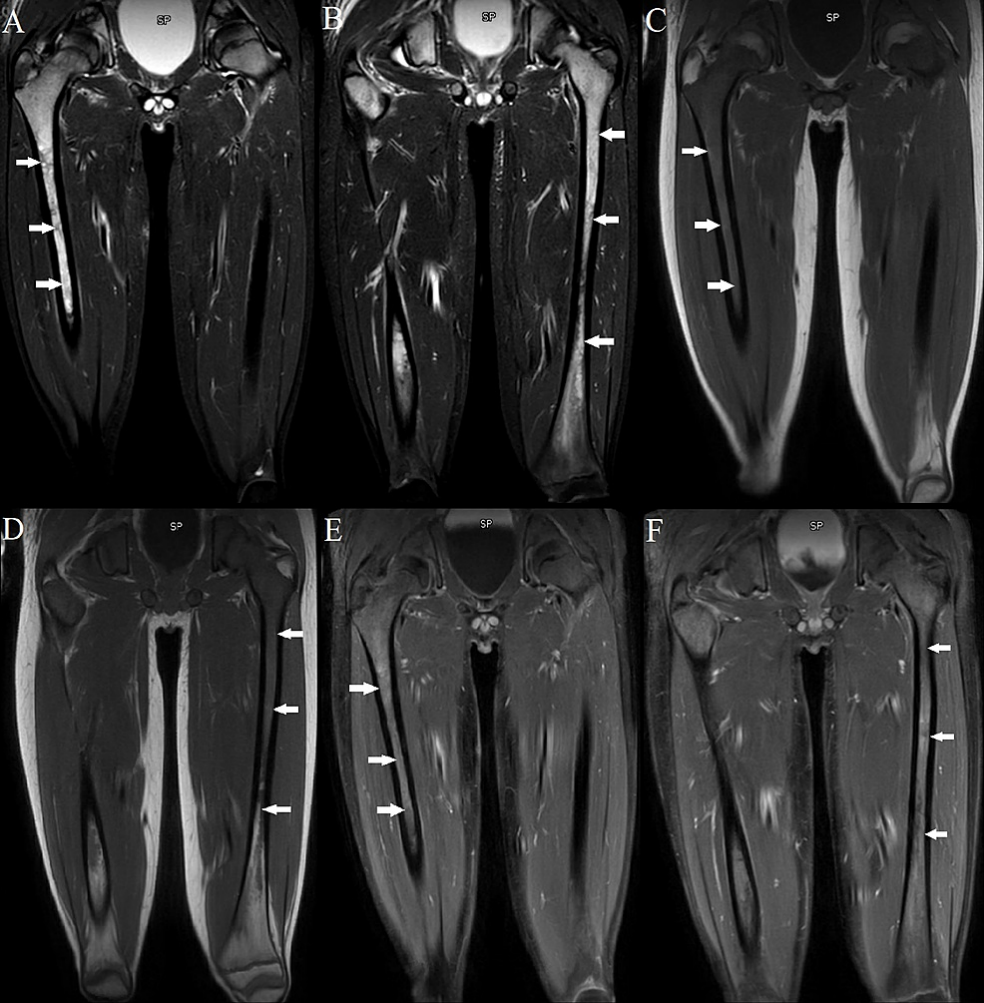
MRI of the lower extremities with and without contrast. (A, B) Coronal STIR sequence. (C, D) Coronal T1 sequence. (E, F) Coronal postcontrast T1 sequence. Note the abnormal marrow signals and heterogenous enhancement involving the bilateral femora in a pattern associated with infiltrative metastatic bone disease.

**Figure 5:**
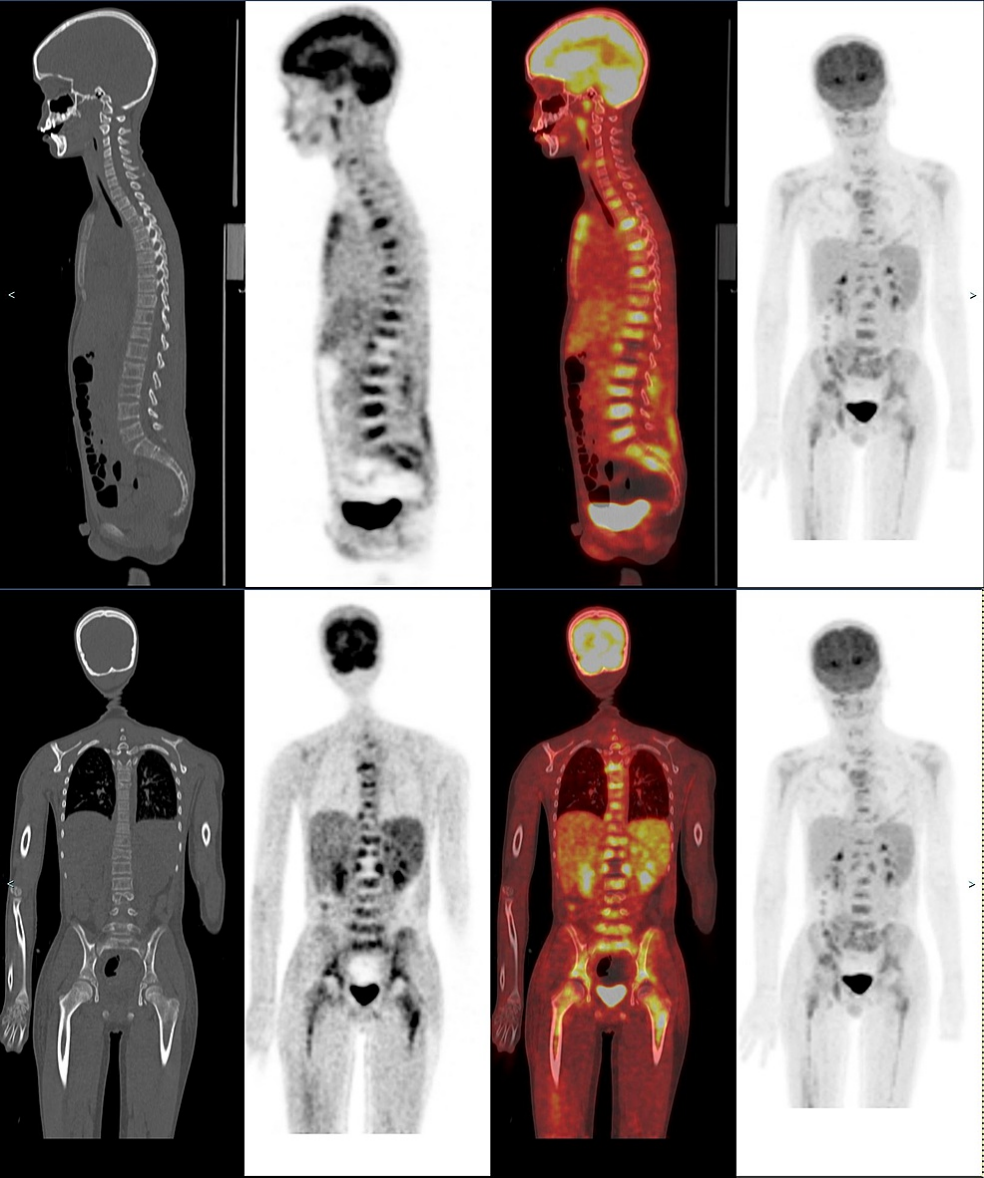
Skull to mid-thigh coronal and sagittal PET-CT (non-enhanced CT, attenuation correction PET slice and fusion images, from left to right) showed diffuse hypermetabolic marrow activity at multiple sites in the spine and pelvis, many of these corresponding to the lytic lesion. Note increased marrow metabolic activity in proximal humeri and femori bilaterally. Maximum intensity projection (MIP) image (last image, from left to right) showed no hypermetabolic focus in cervical, pulmonary regions, and solid abdominal viscera. Overall, these are most concerning for marrow metastatic disease (correlating with prior MRI) without primary site in neck, chest, abdomen, and pelvis.

**Figure 6:**
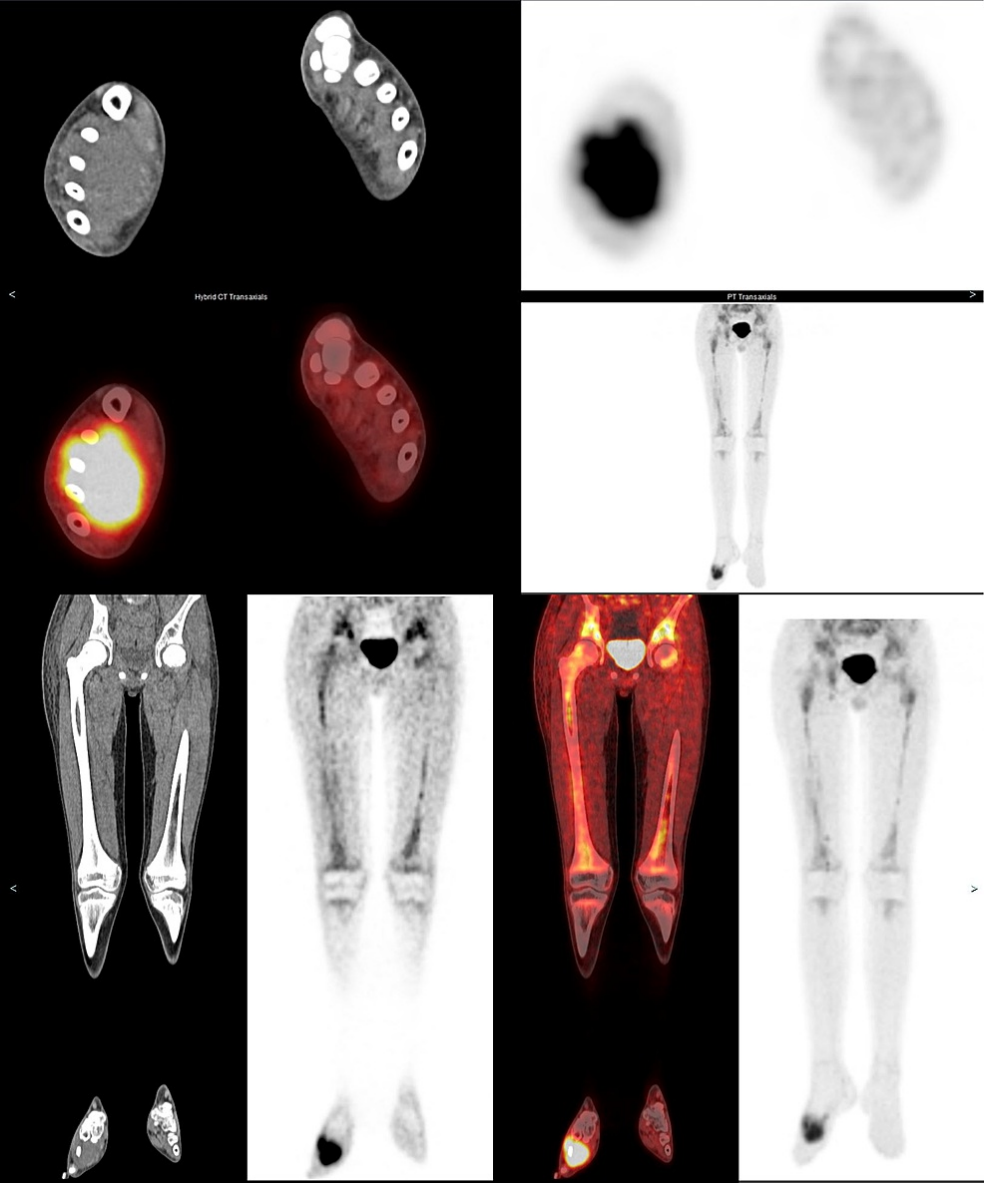
Lower leg axial and coronal PET-CT (non-enhanced CT, attenuation correction PET slice, and fusion images, from left to right) showed hypermetabolic soft tissue mass in the right foot. Coronal PET-CT images and maximum intensity projection (MIP) image (last image, from left to right) confirmed diffuse hypermetabolic marrow in femori bilaterally. Overall, this is concerning for primary mass in the foot with diffuse axial and appendicular marrow metastatic disease.

**Figure 7:**
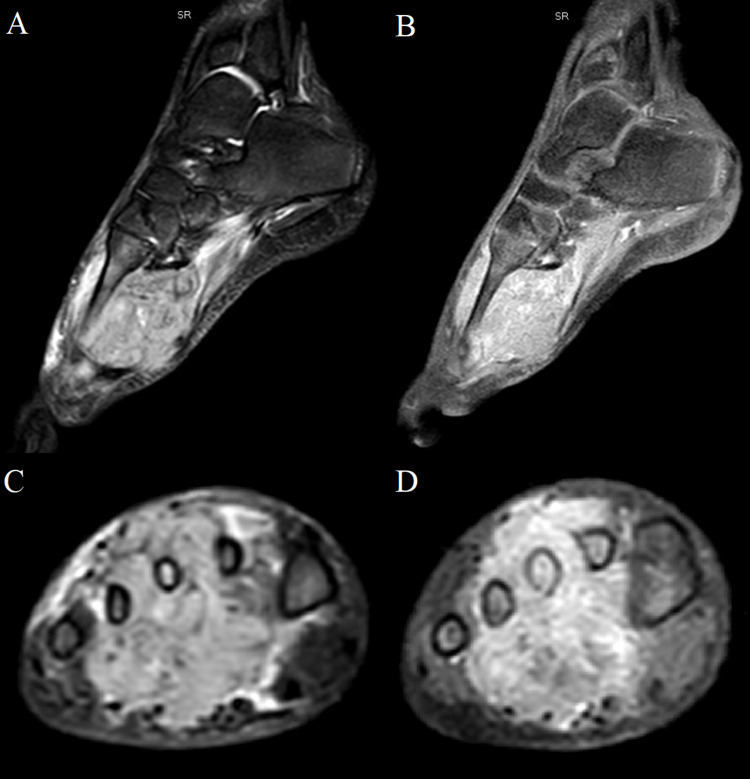
MRI of the right foot with and without contrast. (A) Sagittal STIR sequence. (B) Sagittal postcontrast T1 fat-suppressed sequence. (C) Axial STIR sequence. (D) Axial postcontrast T1 fat-suppressed sequenced. Note the large STIR hyperintense and avidly enhancing soft tissue mass centered in the metatarsal/intermetatarsal region with corresponding marrow signal abnormality.

MRI of the femur, pelvis, and spine was also ordered around this time (Figures 4A-4F). This revealed diffusely abnormal BM signal throughout the visualized axial and appendicular skeleton with multiple compression fractures in the thoracic and lumbar spine. In the lower extremities abnormal marrow signal, heterogeneous enhancement, and restricted diffusion involving the bilateral femurs and pelvic were seen. There was also a conglomeration of enlarged lymph nodes in the groin and popliteal areas noted. However, the site of primary tumor remained undetermined. To evaluate the site of the primary tumor, an MRI of the brain was first placed but found to be negative. Given the negative MRI of the brain, a PET scan was approved for evaluation of the primary tumor site and initial treatment strategies. PET scan showed an ill-defined, hypermetabolic mass in the right foot as well as increased FDG activity of the marrow throughout the axial and appendicular spine consistent with the extensive osseous metastases (Figures 5, 6). An MRI of the tibia, fibula, and foot was also ordered due to the enlarged lymph nodes and revealed a large right foot soft tissue mass with a compatible appearance for RMS (Figures 7A-7D).

At this time, diagnosis of stage IV RMS with primary in the right foot and metastases to the BM was confirmed. Our patient was started on chemotherapy per COG protocol ARST0431.

## Discussion

RMS is the most common childhood and adolescent STS. It is classically classified into two major histopathological subtypes, embryonal RMS (ERMS) (approximately 70%) and ARMS (approximately 20%), however, other subtypes exist including pleomorphic (approximately 10%) and spindle/sclerosing (approximately 10%) [8]. Whereas ERMS is associated with a relatively favorable prognosis, ARMS is typically associated with aggressive behavior, often disseminating throughout the body with a poor response to treatment and frequently relapsing. The alveolar subtype is the most frequent pathologic subtype seen in primary tumors of the extremities. Presenting signs and symptoms are variable and depend on multiple factors including the site of origin, patient’s age, and presence of distant metastases. RMS in the extremities, such as in this case, typically presents as extremity pain, with or without associated erythema of the overlying skin.

While local complications from primary sarcomas can cause significant morbidity, the most life-threatening complications are from the hematogenous dissemination. For most STS originating from the extremity, chest wall, or head and neck, the most common metastatic site is the lungs. Other sites include the BM, liver, breasts, and brain [4]. Approximately 20% of RMS patients will have metastases during the time of diagnosis [8]. However, BM is rarely the sole site of progression or recurrence and is even rarer for it to present without evidence of a primary tumor site [3-6]. In one review of the literature, a total of 39 cases were found with this presentation pattern [9]. BM involvement is typically a poor prognostic sign seen more often in the context of widespread, multifocal metastatic disease [3-4]. When BM involvement appears as the first manifestation of RMS, signs, and symptoms are usually nonspecific including bone and articular pain, fever, nausea, vomiting, lethargy, anorexia, and sometimes anemia and hypercalcemia [4,6].

In cases of RMS with BM metastases presenting with symmetrical diffuse BM involvement, patients can oftentimes be misdiagnosed with leukemia as initially thought in our case. This mimicry can be attributed to the nonspecific findings associated with small primary tumors that do not have overt clinical symptoms at their primary sites [4,9-14]. The origin sometimes remains undetermined after imaging, even postmortem, and may only be diagnosed by BM aspiration [15-16]. In sarcomas with unknown primary sites, misdiagnosis can lead to delayed treatment and increased mortality [4-10]. One case by Imataki et al., which presented as an acute leukemia diagnosis with an initially undetermined primary tumor site, utilized FDG-PET to detect the primary lesion and differentiate RMS from acute leukemia [10]. Interestingly, RMS spread to BM appears related to the presence of certain growth factors such as CCR4 and c-MET in certain tumor populations [17].

In earlier studies, bone scan was the standard for finding osseous metastases. With recent studies though, FDG-PET has been shown to perform better at detecting BM disease during the initial diagnosis of RMS [4,10,15]. There is significant literature support in utilizing FDG-PET as the sole imaging modality for staging of RMS with the elimination of 99mTc-MDP bone scan in most cases [18,19]. The lower detections by bone scans may be due to aggressive osteolysis without osteoblastic response by the metastases [4]. One study by Simmons et al. looked for radiologic features specific to BM metastases from RMS but found no specific diagnostic features. They did note, however, that most deposits were ill-defined lytic lesions with two cases having mixed sclerotic and lytic deposits resulting in an appearance similar to lymphoma [20]. Differentials that must be considered for these atypical cases include non-Hodgkin lymphoma, leukemic infiltration, histiocytosis, metastatic neuroblastoma, Ewing’s sarcoma, and other deposits such as from retinoblastoma [4,15,20]. One diagnostic technique that helps differentiate these tumors is immunohistochemical staining with the most notable marker in differentiating RMS being desmin [5,15].

## Conclusions

In conclusion, the prognosis for RMS with BM metastases is poor and has not substantially improved for the past two decades. While RMS BM metastases traditionally occur in metastatic diseases that have disseminated to multiple parts of the body, there have been reported cases in which BM metastases occur as the sole metastatic location. In some, the primary tumor is not found and can be misdiagnosed as acute leukemia. This concern for myeloproliferative disease can drive physicians to order initial imaging modalities such as MRI targeted towards marrow evaluation to examine the extent of disease. Though in retrospect, MRI may seem unnecessary, this is due to the initial confusing presentation. Once BM aspirate confirms the non-hematologic origin of the neoplasm, PET-CT should be used to detect the origin from an unknown primary. We acknowledge that the costly imaging modalities used in this case are not available worldwide and note this as a limitation in generalizability. This atypical presentation has been limited in radiology literature, however, through our case, we hope to raise awareness of this unique pattern for physicians across all specialties.
